# Supplementary material for: GM-CSF-dependent CD301b+ mouse lung dendritic cells confer tolerance to inhaled allergens
Source: Nat Commun. 2025 Sep 29;16:8547. doi: 10.1038/s41467-025-63547-3 (PMC12480895; doi:10.1038/s41467-025-63547-3)
Supplement: Supplementary file 1 — Supplementary Information [file 41467_2025_63547_MOESM1_ESM.pdf]

Supplementary information

GM-CSF-dependent CD301b^{+} mouse lung dendritic cells confer tolerance to inhaled allergens

Christina L. Wilkinson[1], Keiko Nakano[1], Sara A. Grimm[2], Gregory S. Whitehead[1], Yukitomo Arao[3], Perry J. Blackshear[3], Peer W. Karmaus[1], Michael B. Fessler[1], Donald N. Cook[1] and Hideki Nakano[1]

Corresponding authors;

Hideki Nakano, Ph.D.

Immunity, Inflammation and Disease Laboratory, Division of Intramural Research, National Institute of Environmental Health Sciences, NIH, Research Triangle Park, North Carolina 27709, USA

E-mail: nakanoh@niehs.nih.gov

Donald N. Cook, Ph.D.

Immunity, Inflammation and Disease Laboratory, Division of Intramural Research, National Institute of Environmental Health Sciences, NIH, Research Triangle Park, North Carolina 27709, USA

E-mail: cookd@niehs.nih.gov

Supplementary information contains;

1. Supplementary Figures 1 – 11
2. Supplementary Tables 1
3. References

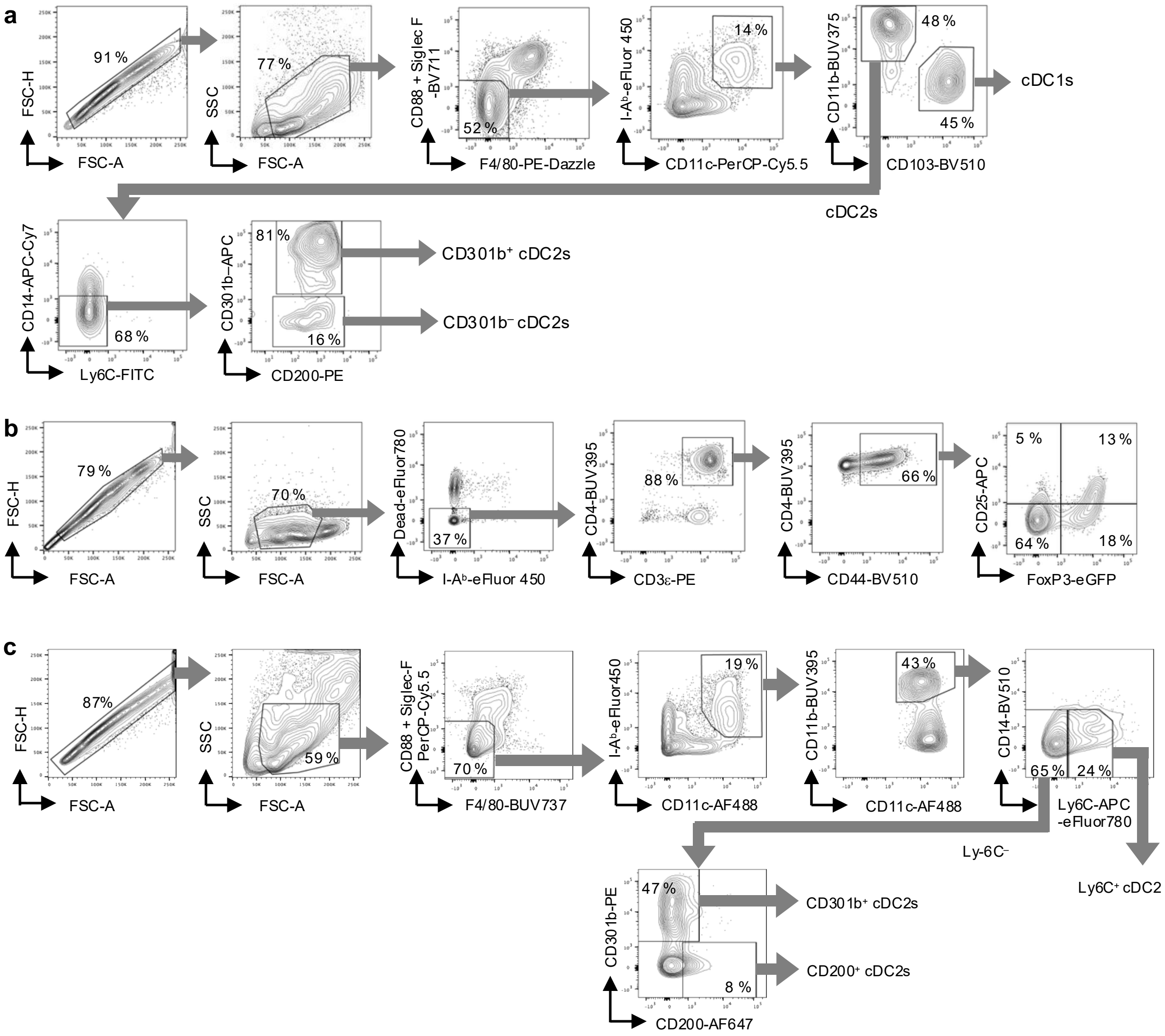

Figure S1 | Gating strategies of cDC2 subset sorting and T cell analysis. a, Gating strategy for sorting of cDC1s and cDC2 subsets. cDC1s: CD11c^{+}I-A^{+}CD11b^{-}CD103^{+}CD88^{-}SiglecF^{-}F4/80^{-}. cDC2s: CD11c^{+}I-A^{+}CD11b^{+}CD103^{-}CD88^{-}SiglecF^{-}F4/80^{-}. The gating strategy was used for Fig. 1c, d and f. **b**, Gating strategy for Treg analysis used for Fig. 1c and d. Tregs were CD4^{+}CD3ε^{+}CD44^{+}I-A^{-}Live/Dead^{-}. **c**, Gating strategy for cDC2 subset purification used for Fig. 1d and Fig. S2c and d. cDC; conventional dendritic cells. Treg; regulatory CD4^{+} T cells.

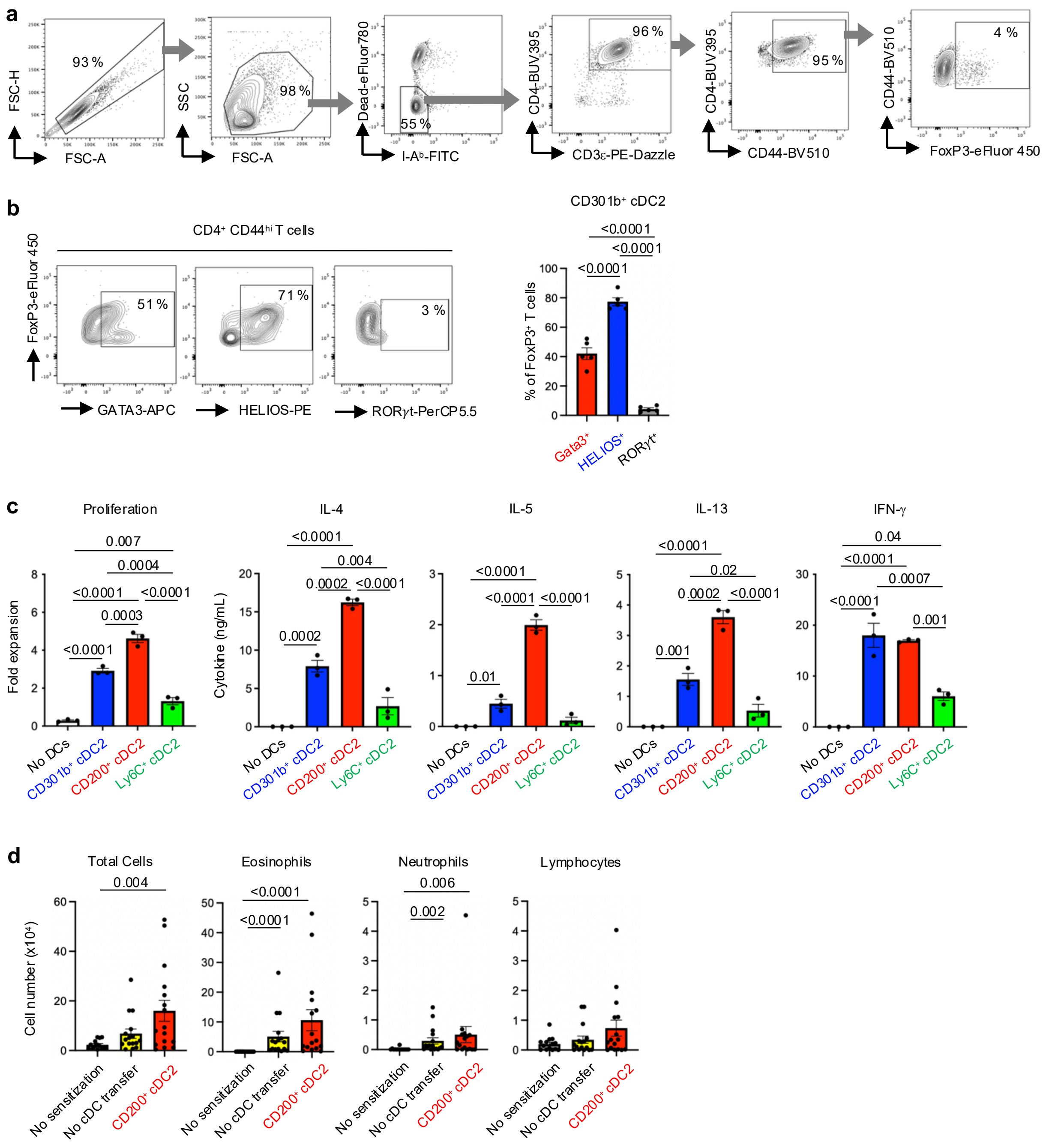

Figure S2 | T cell responses and cDC2-induced responses. a, Gating strategy for Treg analysis in flow cytometry used for Fig. S2b. **b**, Treg transcription factors following OT-II CD4⁺ T cells cocultured with purified CD301b⁺ cDC2s from C57BL/6 mice that received OVA (*n*=5 technical replicates). Representative cytograms (left) compiled data (right). **c**, Proliferation of and cytokine production from CD4⁺ T cells following 5 days culture of naïve CD4⁺ T cells from OT-II mice with the indicated lung cDC2 subsets purified from OVA/HDE-inhaled C57BL/6 mice (*n*=3 technical replicates). **d**, Cell numbers of the indicated leukocytes in BALF following CD200⁺ cDC2 adoptive transfer and OVA/HDE asthma model (No sensitization *n*=14, No cDC transfer *n*=16, CD200⁺ cDC2 *n*=16 biological replicates). Gating strategy of purified cDC2 subsets shown in Fig. S1c. Representative results from two independent experiments (**b, c**) and combined results from three experiments (**d**) are shown. Each dot represents separately cultured CD4⁺ T cells (**b**, **c**) or individual mouse (**d**). (**b, c**) Data were analyzed by one-way ANOVA with Tukey's multiple comparison test. (**d**) Data were analyzed by Kruskal-Wallis test with Dunn's multiple comparisons. Data are presented as mean values ± SEM. *P* values are indicated above the graphs. Source data are provided as a Source Data file, Fig. S2. cDC; conventional dendritic cells. Treg: regulatory CD4⁺ T cells; OVA: ovalbumin; HDE: house dust extract; BALF: bronchoalveolar lavage fluid.

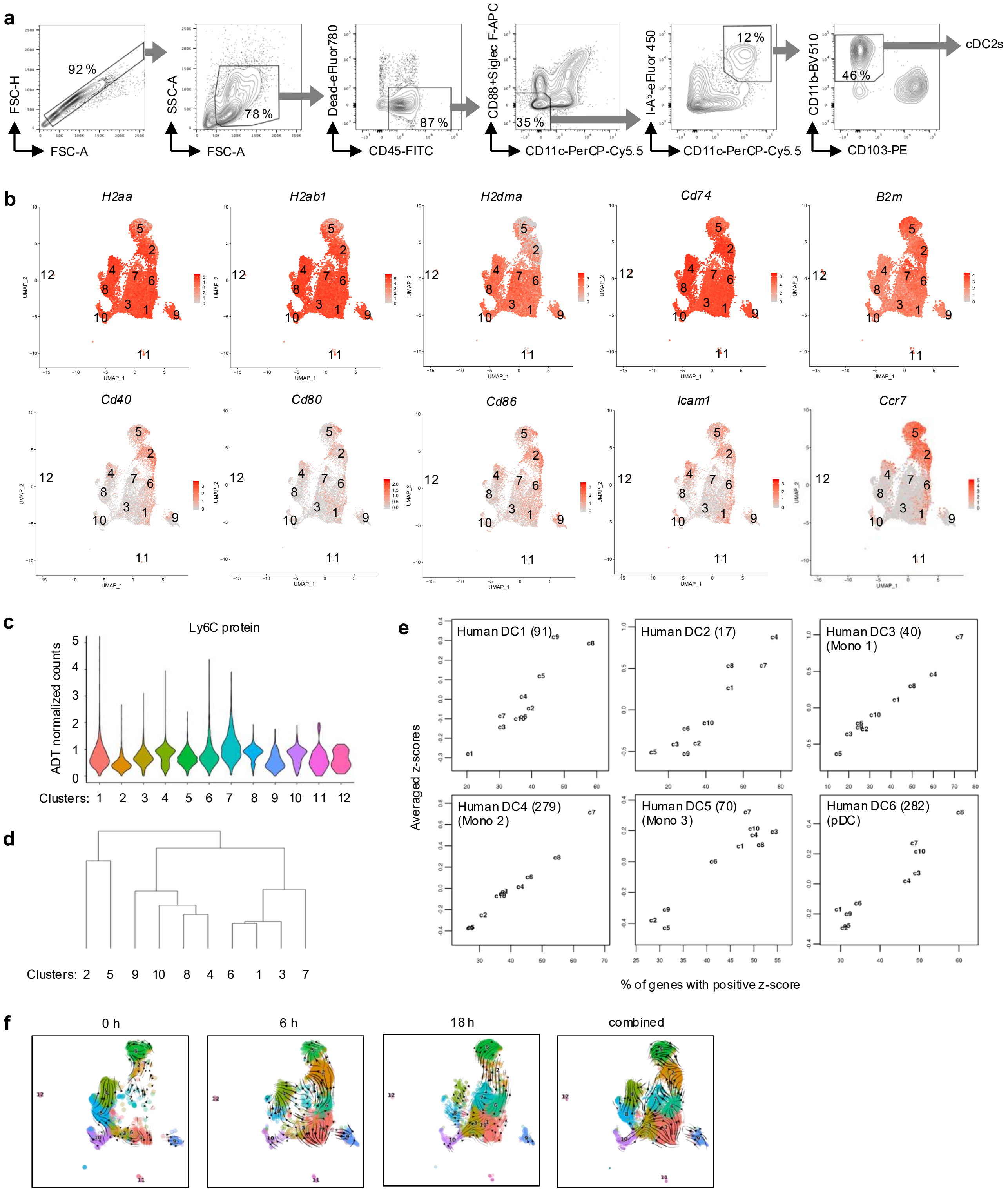

Figure S3 | scRNA-Seq analysis of cDC2s. a, Gating strategy for cDC2 subset sorting for scRNA-sequencing (Fig. 2a-e). **b**, Expression of genes encoding proteins for antigen presentation and costimulation displayed on UMAPs. **c**, Violin plot of Ly6C protein levels on cDC2 clusters analyzed by CITE-Seq. **d**, Similarity of cDC2 clusters was analyzed by Seurat and shown in dendrogram. **e**, Comparison between mouse lung cDC2 clusters with 6 human DC clusters [1]. The numbers of human DC cluster-marker genes used for the analysis are indicated in parentheses. **f**, RNA velocity analysis using stream view (dynamic mode) representing RNA directionality at each time point and combination. cDC: conventional dendritic cells; scRNA-seq: single cell RNA sequencing; UMAP: uniform manifold approximation and projection.

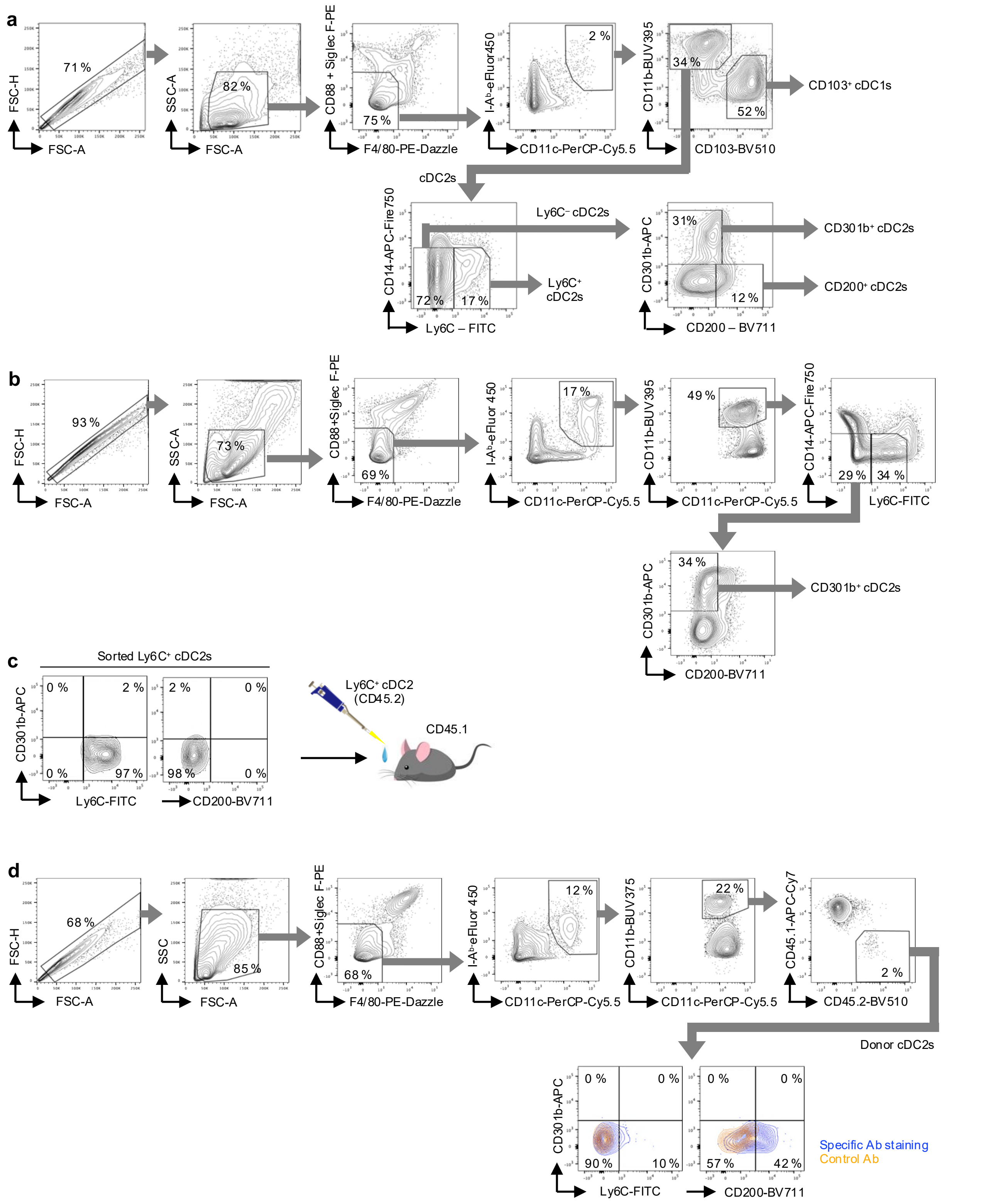

Figure S4 | Gating strategies for cDC2 analysis and adoptive transfer of Ly6C⁺ cDC2s. **a**, Gating strategy for flow cytometric analysis of cDC1 and cDC2 subsets used for Fig. 2f and g. **b**, Gating strategy for CD301b⁺ cDC2 sorting for adoptive transfer in Fig. 2h and Fig. S4c. **c**, Adoptive transfer of purified Ly6C⁺ lung cDC2s from C57BL/6 mice to CD45.1 recipient mice. Representative cytograms of purified donor Ly6C⁺ cDC2s are shown. **d**, Gating strategy for flow cytometry analysis of donor cDC2-derived cells, and cytograms depicting the phenotype of CD45.2⁺ donor-derived Ly6C⁺ cDC2s at 1 day post transfer. Representative results from 2 independent experiments are shown. cDC: conventional dendritic cells.

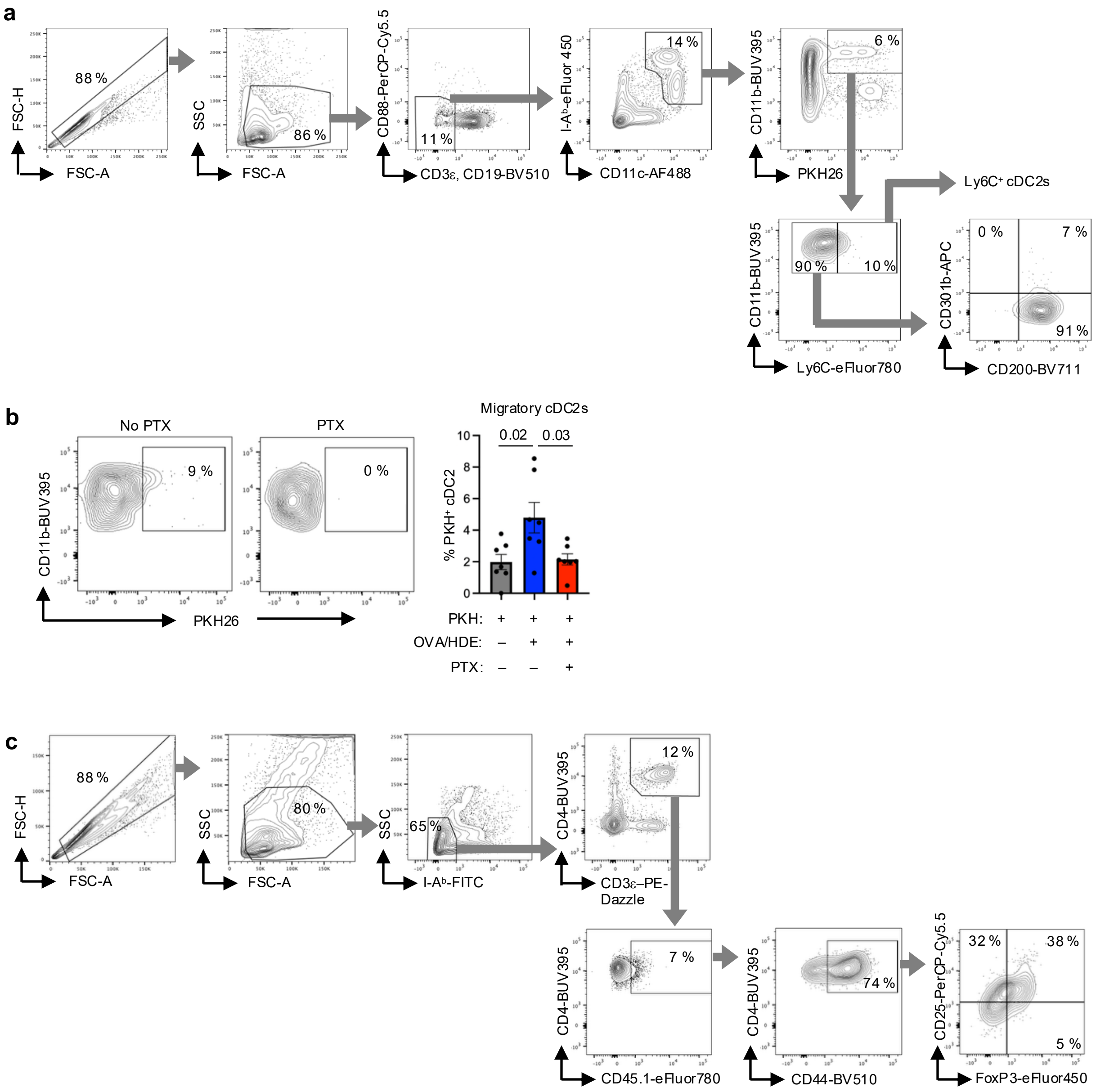

Figure S5 | cDC migration and lung Treg analyses. **a**, Gating strategy for analysis of migratory cDC2 in mLNs in Fig. 3a and b, and Fig. S5b. Phenotype of PKH26+ cells labeled in the lung by dye instillation were analyzed. **b**, DC migration following PTX treatment. Lung cDCs were labeled by o.p. aspiration with PKH, and PKH-positive cDC2s in the mLN following allergic sensitization were detected by flow cytometry (n=7 biological replicates). Each dot represents an individual mouse. Data are presented as mean values ± SEM. Data were analyzed by one-way ANOVA with Tukey's multiple comparisons test. P values are indicated above the graphs. **c**, Gating strategy for Treg analysis used for Fig. 3c and d. Source data are provided as a Source Data file, Fig. S5. cDC: conventional dendritic cells; mLN: mediastinal lymph nodes; PTX: pertussis toxin; Treg: regulatory CD4+ T cells.

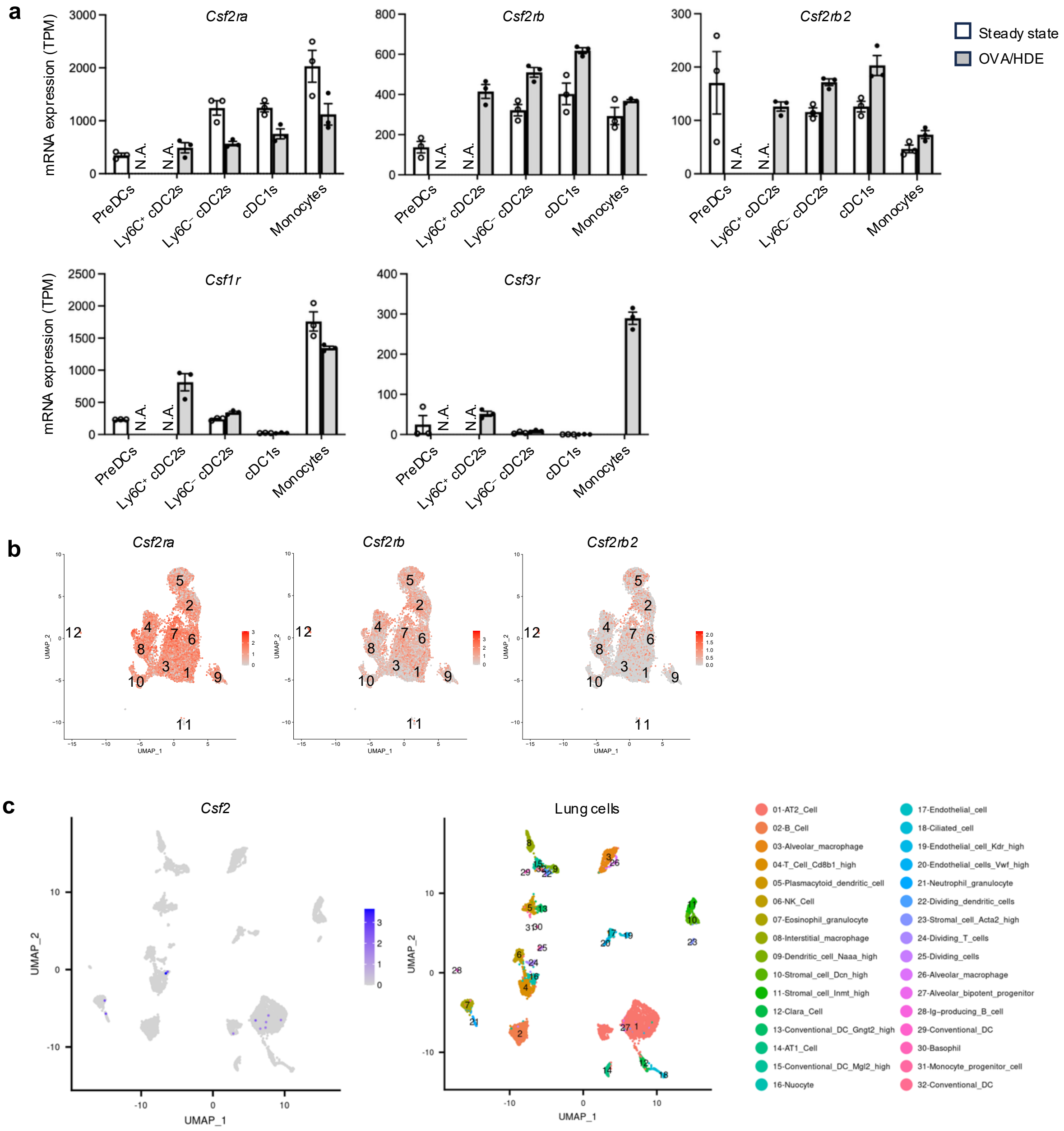

Figure S6 | Expression of genes encoding colony stimulating factor receptors and GM-CSF. a, Expression of *Csf2ra, Csf2rb, Csf2rb2, Csf1r and Csf3r* in lung antigen-presenting cells and bone marrow preDCs at steady state (white) and 16 h after OVA/HDE (grey). Transcripts per million (TPM) of the indicated genes from previously published bulk RNA-Seq data are shown [2]. Each dot represents a biological replicate. **b**, UMAPs of lung cDC2 scRNA-Seq analysis displaying the expression of *Csf2ra, Csf2rb* and *Csf2rb2*. **c**, UMAPs displaying *Csf2* gene expression and lung cell annotation in the analysis of previously published single cell mouse cell atlas data [3]. Source data are provided as a Source Data file, Fig. S6. GM-CSF: granulocyte macrophage colony stimulating factor; preDCs: pre-dendritic cells; scRNA-Seq: single cell RNA sequencing; UMAP: uniform manifold approximation and projection.

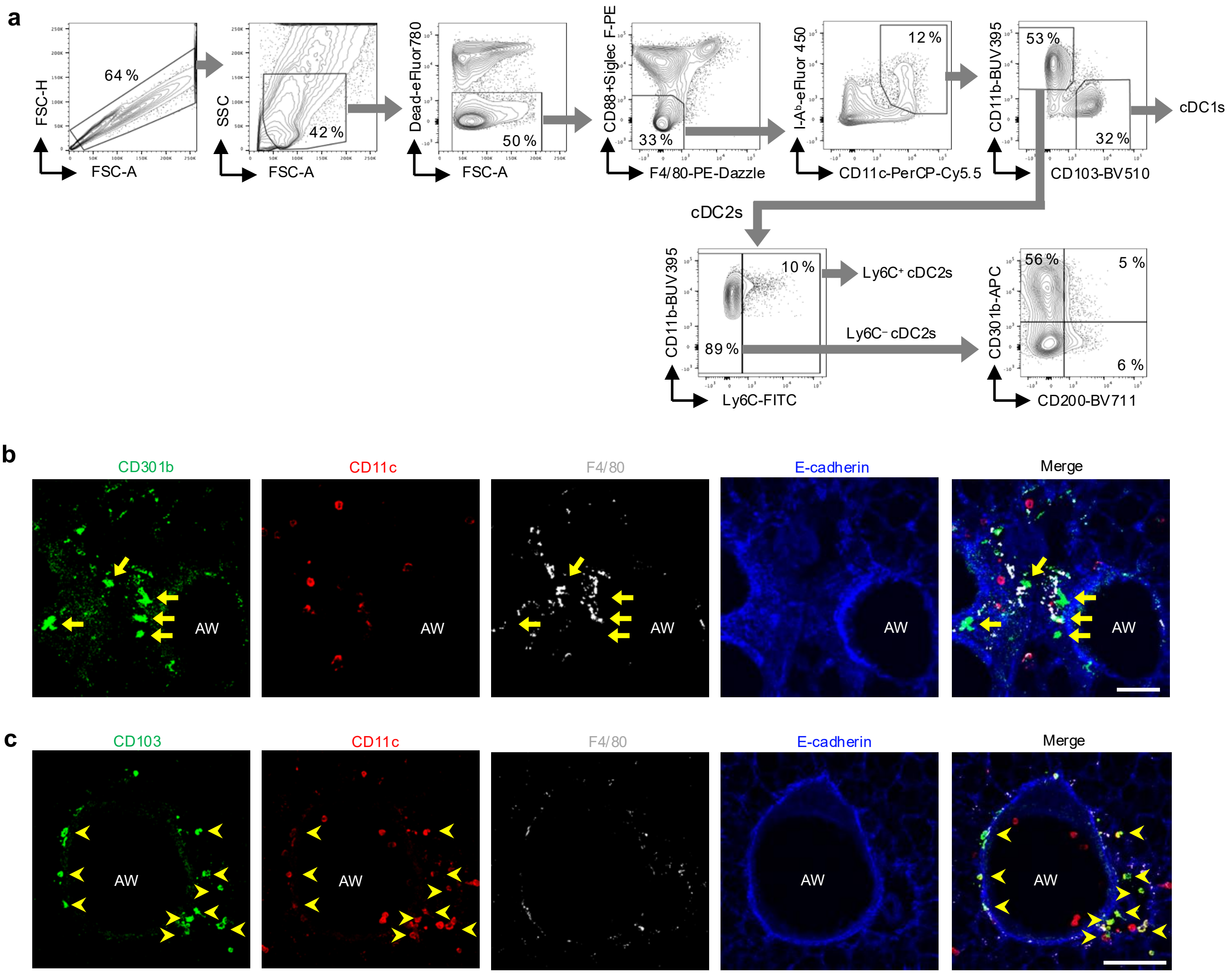

Figure S7 | Gating strategies for mouse lung cDC analysis and localization analysis of lung macrophages and cDC1s. a, Gating strategy for flow cytometry analysis of lung cDC2s from *Csf2*$^{\Delta ARE}$ and *Csf2rb*$^{\Delta DC}$ mice, used for Fig. 4c-f, and Fig. 5a-d. **b**, Representative images of an alveolar airway in PCLS from naïve C57BL/6 mouse lung stained with fluorescent antibodies against CD301b (green), CD11c (red), F4/80 (grey), and E-cadherin (blue). CD301b^{+} macrophages (CD301b^{+}F4/80^{+}CD11c^{-}) are indicated by yellow arrows. **c**, PCLS from naïve C57BL/6 mice stained with fluorescent antibodies against CD103 (green), CD11c (red), F4/80 (grey), and E-cadherin (blue). cDC1s (CD103^{+}CD11c^{+}F4/80^{-}) are indicated by yellow arrow heads. (**b**, **c**) Scale bar represents 50 µm. PCLS: precision cut lung slices; cDC1s: conventional dendritic cell type 1; AW: airway.

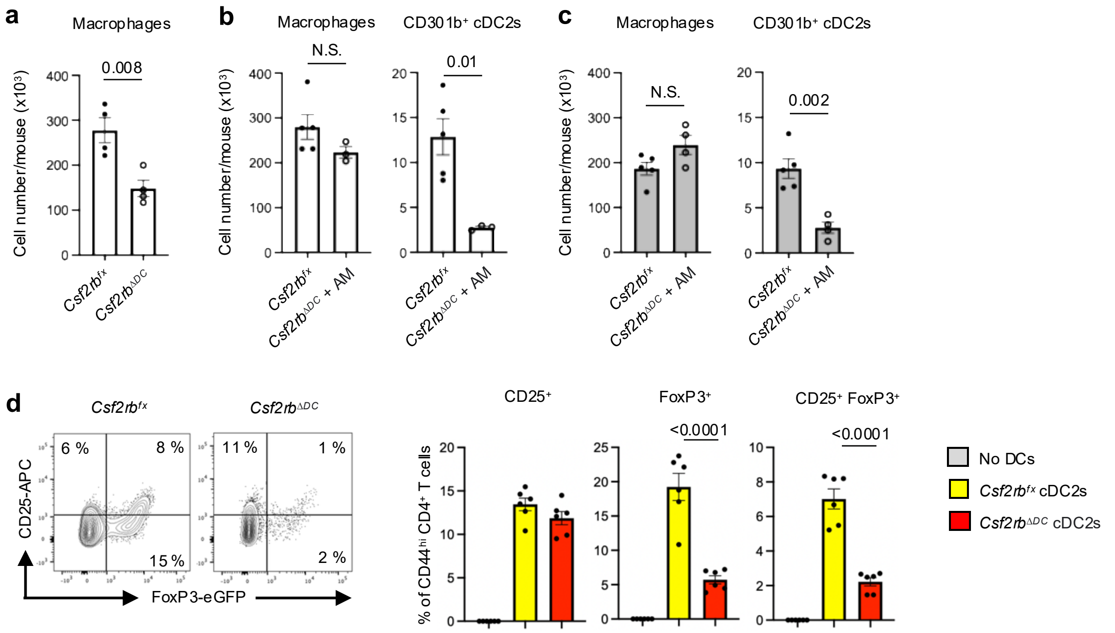

Figure S8 | Reconstitution of alveolar macrophages in *Csf2rb*$^{\Delta DC}$ mice. **a**, The number of lung macrophages in untreated *Csf2rb*^{fx} and *Csf2rb*$^{\Delta DC}$ mice (*n*=4 biological replicates). **b**, **c**, The number of lung macrophages and CD301b⁺ cDC2s at steady state (*Csf2rb*^{fx} *n*=5, *Csf2rb*$^{\Delta DC}$ + AM *n*=3 biological replicates) (**b**) or 16 h post OVA/HDE sensitization (*Csf2rb*^{fx} *n*=5, *Csf2rb*$^{\Delta DC}$ + AM *n*=4 biological replicates) (**c**) in *Csf2rb*^{fx} mice and alveolar macrophage-transferred *Csf2rb*$^{\Delta DC}$ mice. **d**, Treg induction by lung cDC2s. Flow cytometric analysis of CD25⁺ and Foxp3⁺ Tregs after culture of naïve CD4⁺ T cells from *Foxp3*^{eGFP} OT-II mice with or without total lung cDC2s from *Csf2rb*^{fx} mice and alveolar macrophage-transferred *Csf2rb*$^{\Delta DC}$ mice (*n*=6 technical replicates). (**a**-**d**) Representative results from two experiments are shown. Each dot represents individual mouse (**a**-**c**) or separately cultured CD4⁺ T cells (**d**). (**a**-**c**) Data were analyzed by two-tailed unpaired t-test. (**d**) Data were analyzed by one-way ANOVA with Tukey's multiple comparisons test. Data are presented as mean values ± SEM. *P* values are indicated above the graphs. Source data are provided as a Source Data file, Fig. S8. cDC: conventional dendritic cells; OVA: ovalbumin; HDE: house dust extract; Treg: regulatory CD4⁺ T cells.

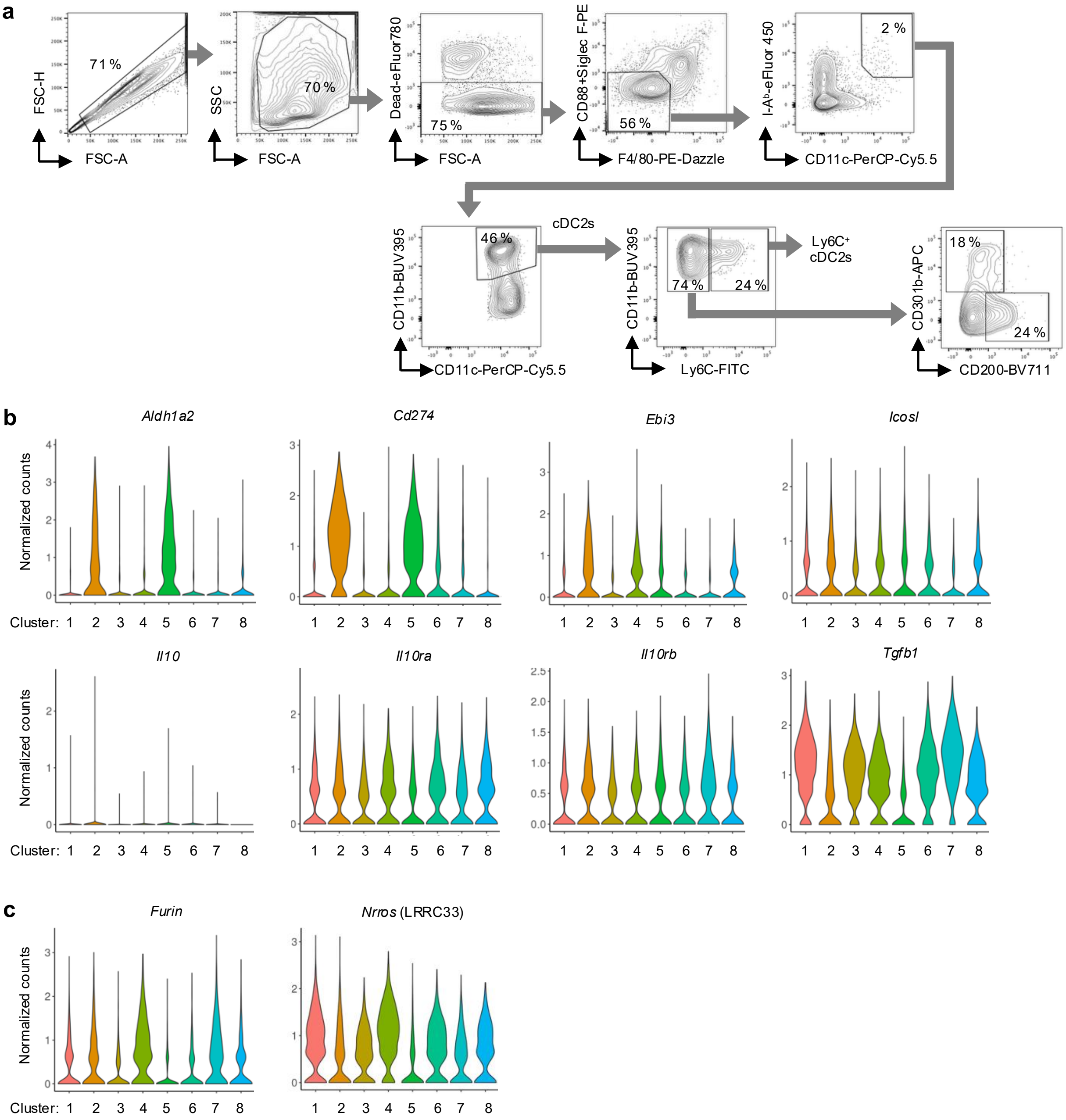

Figure S9 | Expression of genes associated with Treg induction. **a**, Gating strategy for flow cytometric analysis of lung cDC2 subsets used for Fig. 6a. **b**, **c**, Violin plots of lung cDC2 scRNA-Seq analysis displaying expression of genes encoding proteins that are reported to promote Treg differentiation (**b**), and genes encoding FURIN and LRRC33 that are required to activate TGF-β (**c**). cDC: conventional dendritic cells; scRNA-seq: single cell RNA sequencing; Treg; regulatory $CD4^{+}$ T cells; TGF-β: transforming growth factor beta 1.

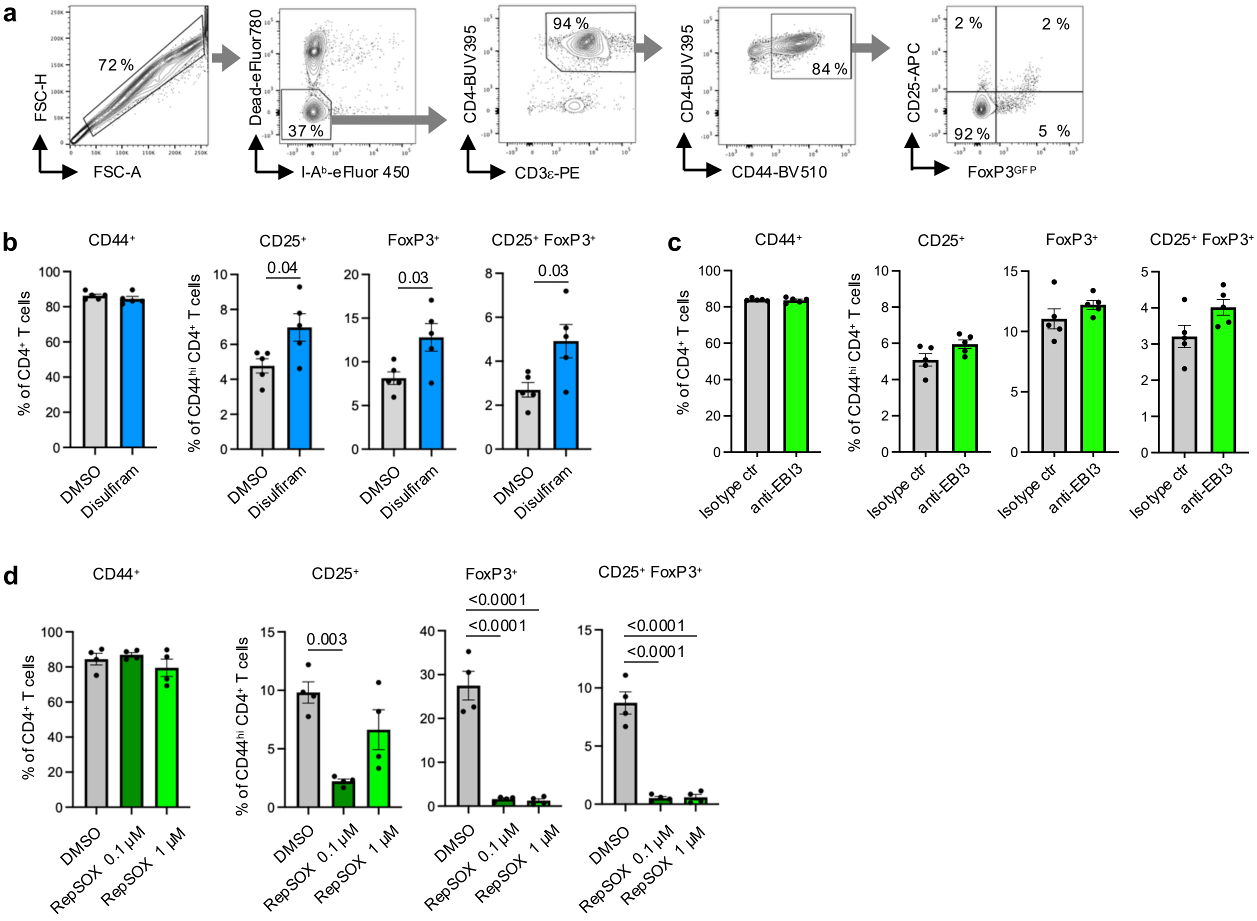

Figure S10 | The effect of inhibitors on Treg induction by lung cDC2s. **a**, Gating strategy for Treg analysis used for Fig. 6c and d, and Fig. S10b-d. **b-d**, Effect of ALDH inhibitor, disulfiram (0.1 μM) (**b**, *n*=5 technical replicates), anti-EBI3 neutralizing antibodies (10 μg/mL) (**c**, *n*=5 technical replicates), or TGF-β type 1 receptor/ALK5 inhibitor (RepSOX) at indicated concentration (**d**, *n*=4 technical replicates), on Treg induction by lung cDC2s. Naïve CD4+ T cells from *Foxp3eGFP* OT-II mice were cultured with total cDC2s from C57BL/6 mice for 5 days and analyzed by flow cytometry. Each dot represents a separate culture of CD4+ T cells. Data were analyzed by two-tailed unpaired *t*-test (**b**, **c**) or by one-way ANOVA with Tukey's multiple comparison test (**d**). Representative results from 2 independent experiments are shown. Data are presented as mean values ± SEM. *P* values are indicated above the graphs. Source data are provided as a Source Data file, Fig. S10. cDC: conventional dendritic cells; Treg: regulatory CD4+ T cells; ALDH: aldehyde dehydrogenase; EBI3: Epstein-Barr virus induced 3; TGF-β: transforming growth factor beta 1; Treg: regulatory CD4+ T cells.

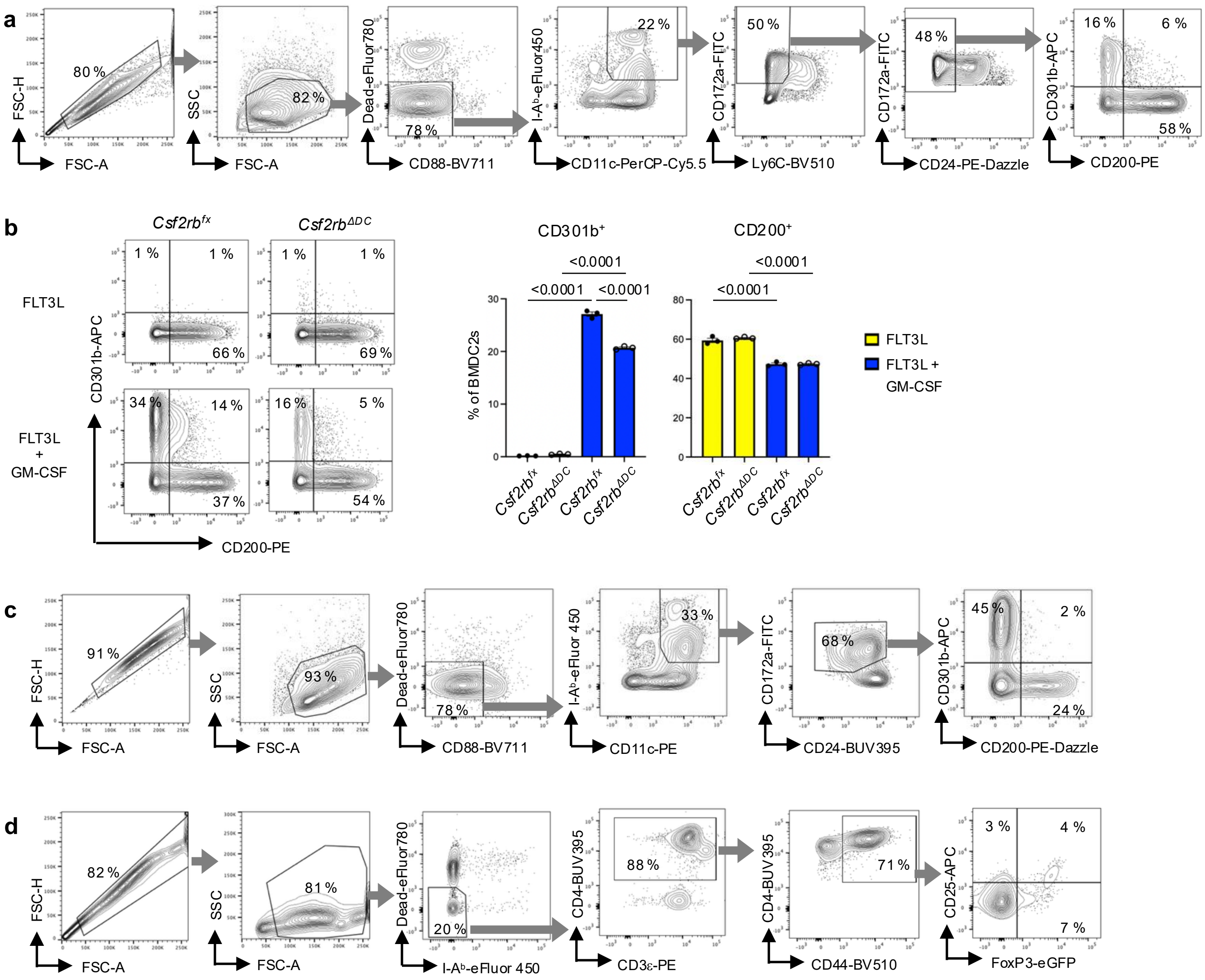

Figure S11 | Gating strategies for BMDC development and Treg induction. **a**, Gating strategy for BMDC2 analysis used for Fig. 7a. **b**, BMDC2s were generated from *Csf2rb*^{fx} or *Csf2rb*$^{\Delta DC}$ mice by *in vitro* culture of bone marrow cells with FLT3L for 6 days, and further cultured with or without GM-CSF for 24 hrs. Representative cytograms (left panels) and compiled data (right panels) of flow cytometric analysis of BMDC2s (CD11c$^+$I-A$^+$CD172a$^+$Ly6C$^-$) are shown (*n*=3 technical replicates). Each dot represents separately cultured bone marrow cells. Representative results from two independent experiments are shown. Data are presented as mean values ± SEM. Data were analyzed by one-way ANOVA with Tukey's multiple comparison test. *P* values are indicated above the graphs. **c**, Gating strategy for CD301b$^+$ and CD200$^+$ BMDC2 purification used for Fig. 7b-e. **d**, Gating strategy for flow cytometric analysis of Tregs following *ex vivo* culture used for Fig. 7c. Source data are provided as a Source Data file, Fig. S11. BMDC: bone marrow dendritic cells; Treg: regulatory CD4$^+$ T cells; FLT3L: FMS-like tyrosine kinase 3 ligand; GM-CSF: granulocyte macrophage colony stimulating factor.

Supplementary Table 1. Antibodies used in this study

Antibody	Clone	Source	Catalog #	Concentration (μg/mL)	Use in study
CD200	OX-90	BioLegend	123811	10	CITE-Seq
CD301b	URA-1	BioLegend	146817	10	CITE-Seq
Ly6C	HK1.4	BioLegend	128047	10	CITE-Seq
AF488-anti-mouse CD11c	N418	eBioscience	53-0114-82	1	FC
AF647-anti-mouse CD200	OX-90	BioLegend	123816	1	FC
AF700-anti-mouse CD116/CSF2RA	698423	R&D Systems	FAB6130N	1	FC
APC eF780-anti-mouse CD14	Sa14-2	BioLegend	123331	1	FC
APC eFluor 780-anti-mouse Ly-6C	HK1.4	eBioscience	47-5932-82	1	FC
APC-anti-mouse CD25	3C7	BioLegend	101910	1	FC
APC-anti-mouse CD301b	URA-1	BioLegend	146813	1	FC
APC-anti-mouse CD88	20/70	BioLegend	135808	1	FC
APC-anti-mouse GATA3	W19195B	BioLegend	386908	0.5	FC
AF647-anti-mouse Siglec-F	E50-2440	BD	562680	1	FC
APC-Cy7-anti-mouse CD45.1	A20	BioLegend	110716	1	FC
APC-rat IgG2aκ	eBR2a	eBioscience	17-4321-81	1	FC
APC-rat IgG2aκ	RTK2758	BioLegend	400511	0.5	FC
BUV395-anti-mouse CD11b	M1/70	BD	565553	1	FC
BUV395-anti-mouse CD24	M1/69	BD	744471	1	FC
BUV395-anti-mouse CD4	RM4-4	BD	740209	1	FC
BUV395-anti-mouse CD86	P03	BD	745716	1	FC
BUV737-anti-mouse F4/80	T45-2342	BD	749283	1	FC
BV510-anti-mouse CD103	M290	BD	563087	1	FC
BV510-anti-mouse CD11b	M1/70	BioLegend	101263	1	FC
BV510-anti-mouse CD14	Sa14-2	BioLegend	123323	1	FC
BV510-anti-mouse CD44	IM7	BioLegend	103044	1	FC
BV510-anti-mouse CD45.2	104	BioLegend	109837	1	FC
BV510-anti-mouse Ly-6C	HK1.4	BioLegend	128033	1	FC
BV650-anti-mouse IgG1κ	R19-15	BD	744532	0.5	FC
BV650-anti-mouse RORγt	Q31-378	BD	564722	0.5	FC
BV711-anti-mouse CD200	OX-90	BD	745548	1	FC
BV711-anti-mouse CD88	20/70	BD	743773	1	FC
BV711-anti-mouse Ly-6A/E	D7	BioLegend	108131	0.5	FC
BV711-anti-mouse Siglec-F	E50-2440	BD	740784	0.25	FC
BV711-rat IgG2a	RTK2758	BioLegend	400551	1	FC
eFluo450-anti-mouse FoxP3	FJK-16s	eBioscience	48-5773-82	2	FC
eFluor450-anti-mouse MHC-II I-Ab	AF6-120.1	eBioscience	48-5320-82	1	FC
eFluor450-rat IgG2aκ	eBR2a	eBioscience	48-4321-82	2	FC
FITC-anti-mouse CD172a	P84	BD	560316	2.5	FC
FITC-anti-mouse CD40	3/23	BD	561845	1	FC
FITC-anti-mouse CD45	30-F11	BioLegend	103108	1	FC

FITC-anti-mouse CD80	16-10A1	eBioscience	11-0801-82	1	FC
FITC-anti-mouse CD86	GL1	BD	561962	1	FC
FITC-anti-mouse Ly-6C	AL-21	BD	553104	2.5	FC
FITC-rat IgG2a	R35-95	BD	554688	1	FC
FITC-rat IgMκ	RTK2118	BioLegend	400805	2.5	FC
PE-anti-hamster IgG	HTK888	BioLegend	400907	1	FC
PE-anti-mouse CD131/CSF2RB	REA193	BD	559920	1	FC
PE-anti-mouse CD200	OX-90	BioLegend	123807	0.5	FC
PE-anti-mouse CD301b	URA-1	BioLegend	146804	1	FC
PE-anti-mouse CD3e	145-2C11	BioLegend	100307	1	FC
PE-anti-mouse CD88	20/70	BioLegend	135806	1	FC
PE-anti-mouse HELIOS	22F6	BioLegend	137206	1	FC
PE-anti-mouse Siglec-F	S17007L	BD	552126	0.5	FC
PE-Dazzle-anti-mouse CD24	M1/69	BioLegend	101837	1	FC
PE-Dazzle594-anti-mouse F4/80	BM8	BioLegend	123146	1	FC
PE-rat IgG2a	RTK2758	BioLegend	400508	0.5	FC
PE-rat IgG2b	eB149/10H5	eBioscience	12-4031-82	0.5	FC
PerCP-Cy5.5-anti-mouse CD11c	N418	BioLegend	117328	1	FC
PerCP-Cy5.5-anti-mouse CD45RB	C363-16A	BioLegend	103313	1	FC
PerCP-Cy5.5-anti-mouse CD88	20/70	BioLegend	135813	1	FC
Biotin-anti-mouse CD11b	M1/70	BD	553309	0.5	MACS
Biotin-anti-mouse CD11c	N418	BioLegend	117304	0.5	MACS
Biotin-anti-mouse CD16/32	2.4G2	BD	553143	0.5	MACS
Biotin-anti-mouse CD19	6D5	BioLegend	115504	0.5	MACS
Biotin-anti-mouse CD25	PC61	BioLegend	102004	0.5	MACS
Biotin-anti-mouse CD44	IM7	BioLegend	103004	0.05	MACS
Biotin-anti-mouse CD49b	DX5	BD	553856	0.5	MACS
Biotin-anti-mouse CD8α	53-6.7	BD	553029	0.5	MACS
Biotin-anti-mouse CD8β	53-5.8	BD	553039	0.5	MACS
Biotin-anti-mouse I-Ab	AF6.120.1	BioLegend	116404	0.5	MACS
Biotin-anti-mouse Ly-6C/G	RB6-8C5	BD	553125	0.5	MACS
Purified-anti-mouse CD28	37.51	BioLegend	102116	1	MACS
Purified-anti-mouse CD3e	145-2C11	BioLegend	100331	1	MACS
Anti-EBI3/IL-35	V1.4C4.22	Sigma	MABF848	10	Neutralization
AF488-anti-mouse CD324	DECMA-1	Invitrogen	560061	1	PCLS
APC-anti-mouse CD103	2E7	BioLegend	121414	1	PCLS
APC-anti-mouse CD301b	URA-1	BioLegend	146814	1	PCLS
BV605-anti-mouse CD11c	N418	BioLegend	117334	1	PCLS
PE-anti-mouse F4/80	BM8	BioLegend	123110	1	PCLS

CITE-Seq: cellular indexing of transcriptomes and epitopes by sequencing, FC: flow cytometry, MACS: magnetic-activated cell sorting, PCLS: precision cut lung slices.

References

1. Villani, A.C. *et al.* Single-cell RNA-seq reveals new types of human blood dendritic cells, monocytes, and progenitors. *Science* **356** (2017).

2. Izumi, G. *et al.* CD11b(+) lung dendritic cells at different stages of maturation induce Th17 or Th2 differentiation. *Nat Commun* **12**, 5029 (2021).

3. Han, X. *et al.* Mapping the Mouse Cell Atlas by Microwell-Seq. *Cell* **172**, 1091-1107 e1017 (2018).
