# Supplementary material for: GM-CSF-dependent CD301b+ mouse lung dendritic cells confer tolerance to inhaled allergens
Source: Nat Commun. 2025 Sep 29;16:8547. doi: 10.1038/s41467-025-63547-3 (PMC12480895; doi:10.1038/s41467-025-63547-3)
Supplement: Supplementary file 2 — Description of Additional Supplementary Files [file 41467_2025_63547_MOESM2_ESM.pdf]

Description of Additional Supplementary Files

Supplementary Data 1: Seurat code for scRNA-Seq UMAP

Supplementary Data 2: scRNA-Seq RNA velocity analysis 0h

Supplementary Data 3: scRNA-Seq RNA velocity analysis 6h

Supplementary Data 4: scRNA-Seq RNA velocity analysis 18h

Supplementary Data 5: scRNA-Seq RNA velocity analysis combined
