# Supplementary material for: GM-CSF-dependent CD301b+ mouse lung dendritic cells confer tolerance to inhaled allergens
Source: Nat Commun. 2025 Sep 29;16:8547. doi: 10.1038/s41467-025-63547-3 (PMC12480895; doi:10.1038/s41467-025-63547-3)
Supplement: Supplementary file 8 — Reporting Summary [file 41467_2025_63547_MOESM8_ESM.pdf]

nature portfolio

Corresponding author(s): Hideki Nakano

Last updated by author(s): Jul 10, 2025

Reporting Summary

Nature Portfolio wishes to improve the reproducibility of the work that we publish. This form provides structure for consistency and transparency in reporting. For further information on Nature Portfolio policies, see our Editorial Policies and the Editorial Policy Checklist.

Statistics

For all statistical analyses, confirm that the following items are present in the figure legend, table legend, main text, or Methods section.

n/a	Confirmed	
☐	☒	The exact sample size (*n*) for each experimental group/condition, given as a discrete number and unit of measurement
☐	☒	A statement on whether measurements were taken from distinct samples or whether the same sample was measured repeatedly
☐	☒	The statistical test(s) used AND whether they are one- or two-sided<br>*Only common tests should be described solely by name; describe more complex techniques in the Methods section.*
☐	☒	A description of all covariates tested
☐	☒	A description of any assumptions or corrections, such as tests of normality and adjustment for multiple comparisons
☐	☒	A full description of the statistical parameters including central tendency (e.g. means) or other basic estimates (e.g. regression coefficient) AND variation (e.g. standard deviation) or associated estimates of uncertainty (e.g. confidence intervals)
☐	☒	For null hypothesis testing, the test statistic (e.g. *F*, *t*, *r*) with confidence intervals, effect sizes, degrees of freedom and *P* value noted<br>*Give P values as exact values whenever suitable.*
☒	☐	For Bayesian analysis, information on the choice of priors and Markov chain Monte Carlo settings
☒	☐	For hierarchical and complex designs, identification of the appropriate level for tests and full reporting of outcomes
☒	☐	Estimates of effect sizes (e.g. Cohen's *d*, Pearson's *r*), indicating how they were calculated

Our web collection on statistics for biologists contains articles on many of the points above.

Software and code

Policy information about availability of computer code

Data collection	Flow cytometry sorting: FACS Diva version 8.0.1 (BD)<br>Flow cytometry analysis: FACS Diva version 8.0.2 (BD)<br>ELISA: Ascent version 2.6 (Thermo Electron)<br>Microscopy: Zen Blue version 3.9.3 (Zeiss)<br>scRNA-Seq: RTA version 2.4.11 (Illumina)
Data analysis	GraphPad Prism version 9.5.1 and 10.4.1 (GraphPad)<br>Flow cytometry: Flow Jo version 10.8.1 and 10.8.2 (BD)<br>scRNA-Seq: Cell Ranger 3.1.0 (10X Genomics), Seurat version 3.0 package in R version 3.6.2 (http://satijalab.org/seurat/), scVelo version 0.2.4 (https://scvelo.readthedocs.io/en/stable/)

For manuscripts utilizing custom algorithms or software that are central to the research but not yet described in published literature, software must be made available to editors and reviewers. We strongly encourage code deposition in a community repository (e.g. GitHub). See the Nature Portfolio guidelines for submitting code & software for further information.

Data

Policy information about availability of data

All manuscripts must include a data availability statement. This statement should provide the following information, where applicable:

- Accession codes, unique identifiers, or web links for publicly available datasets
- A description of any restrictions on data availability
- For clinical datasets or third party data, please ensure that the statement adheres to our policy

Data availability has been described in the Data availability section in the manuscript. CD11b+ cDC2 CITE-Seq data generated in this study have been deposited in GEO under accession code GSE261034. Other scRNA-Seq (GEO accession code GSE108097) and bulk RNA-Seq (GEO accession code GSE149778) data from previously published papers were also used in this study18, 35. The raw data used for generating graphs presented in this manuscript are provided in a Source Data file.

Research involving human participants, their data, or biological material

Policy information about studies with human participants or human data. See also policy information about sex, gender (identity/presentation), and sexual orientation and race, ethnicity and racism.

Reporting on sex and gender	*Use the terms sex (biological attribute) and gender (shaped by social and cultural circumstances) carefully in order to avoid confusing both terms. Indicate if findings apply to only one sex or gender; describe whether sex and gender were considered in study design; whether sex and/or gender was determined based on self-reporting or assigned and methods used.* *Provide in the source data disaggregated sex and gender data, where this information has been collected, and if consent has been obtained for sharing of individual-level data; provide overall numbers in this Reporting Summary. Please state if this information has not been collected.* *Report sex- and gender-based analyses where performed, justify reasons for lack of sex- and gender-based analysis.*
Reporting on race, ethnicity, or other socially relevant groupings	*Please specify the socially constructed or socially relevant categorization variable(s) used in your manuscript and explain why they were used. Please note that such variables should not be used as proxies for other socially constructed/relevant variables (for example, race or ethnicity should not be used as a proxy for socioeconomic status).* *Provide clear definitions of the relevant terms used, how they were provided (by the participants/respondents, the researchers, or third parties), and the method(s) used to classify people into the different categories (e.g. self-report, census or administrative data, social media data, etc.)* *Please provide details about how you controlled for confounding variables in your analyses.*
Population characteristics	*Describe the covariate-relevant population characteristics of the human research participants (e.g. age, genotypic information, past and current diagnosis and treatment categories). If you filled out the behavioural & social sciences study design questions and have nothing to add here, write "See above."*
Recruitment	*Describe how participants were recruited. Outline any potential self-selection bias or other biases that may be present and how these are likely to impact results.*
Ethics oversight	*Identify the organization(s) that approved the study protocol.*

Note that full information on the approval of the study protocol must also be provided in the manuscript.

Field-specific reporting

Please select the one below that is the best fit for your research. If you are not sure, read the appropriate sections before making your selection.

☐ Life sciences ☐ Behavioural & social sciences ☐ Ecological, evolutionary & environmental sciences

For a reference copy of the document with all sections, see nature.com/documents/nr-reporting-summary-flat.pdf

Life sciences study design

All studies must disclose on these points even when the disclosure is negative.

Sample size	A power calculation estimated that n=5 per group to detect differences between groups for in vivo mouse model asthma experiments with power value 0.8 and significance level 0.05. Sample size was determined to obtain reliable statistical results based on experience from previous studies. For ex vivo flow cytometric analysis, n=3 replicates per group detect differences between groups. For in vitro experiments, n=3 technical replicates per group were sufficient to detect differences between groups. Sample size of each graph is provided in figure legend.
Data exclusions	Outliers were identified by Prism software using Identify Outlier function with ROUT method and Q=1% aggressiveness. This criterion was pre-established in the software. We excluded data identified as outliers by Prism.
Replication	Experiments were performed at least twice except for CITE-Seq. The number of experimental repeats is shown in the figure legends.

Randomization	Age- and sex-matched wild type and knockout or transgenic mice were assigned to different groups. Mice were randomly allocated into control or treatment groups.
Blinding	Experiments were not performed in a blind manner. Genotyping was necessary to allow identification of mice of the appropriate age, gender, and genotype. Thus, investigators were aware of the genotyping results prior to beginning the experiments.

Behavioural & social sciences study design

All studies must disclose on these points even when the disclosure is negative.

Study description	*Briefly describe the study type including whether data are quantitative, qualitative, or mixed-methods (e.g. qualitative cross-sectional, quantitative experimental, mixed-methods case study).*
Research sample	*State the research sample (e.g. Harvard university undergraduates, villagers in rural India) and provide relevant demographic information (e.g. age, sex) and indicate whether the sample is representative. Provide a rationale for the study sample chosen. For studies involving existing datasets, please describe the dataset and source.*
Sampling strategy	*Describe the sampling procedure (e.g. random, snowball, stratified, convenience). Describe the statistical methods that were used to predetermine sample size OR if no sample-size calculation was performed, describe how sample sizes were chosen and provide a rationale for why these sample sizes are sufficient. For qualitative data, please indicate whether data saturation was considered, and what criteria were used to decide that no further sampling was needed.*
Data collection	*Provide details about the data collection procedure, including the instruments or devices used to record the data (e.g. pen and paper, computer, eye tracker, video or audio equipment) whether anyone was present besides the participant(s) and the researcher, and whether the researcher was blind to experimental condition and/or the study hypothesis during data collection.*
Timing	*Indicate the start and stop dates of data collection. If there is a gap between collection periods, state the dates for each sample cohort.*
Data exclusions	*If no data were excluded from the analyses, state so OR if data were excluded, provide the exact number of exclusions and the rationale behind them, indicating whether exclusion criteria were pre-established.*
Non-participation	*State how many participants dropped out/declined participation and the reason(s) given OR provide response rate OR state that no participants dropped out/declined participation.*
Randomization	*If participants were not allocated into experimental groups, state so OR describe how participants were allocated to groups, and if allocation was not random, describe how covariates were controlled.*

Ecological, evolutionary & environmental sciences study design

All studies must disclose on these points even when the disclosure is negative.

Study description	*Briefly describe the study. For quantitative data include treatment factors and interactions, design structure (e.g. factorial, nested, hierarchical), nature and number of experimental units and replicates.*
Research sample	*Describe the research sample (e.g. a group of tagged Passer domesticus, all Stenocereus thurberi within Organ Pipe Cactus National Monument), and provide a rationale for the sample choice. When relevant, describe the organism taxa, source, sex, age range and any manipulations. State what population the sample is meant to represent when applicable. For studies involving existing datasets, describe the data and its source.*
Sampling strategy	*Note the sampling procedure. Describe the statistical methods that were used to predetermine sample size OR if no sample-size calculation was performed, describe how sample sizes were chosen and provide a rationale for why these sample sizes are sufficient.*
Data collection	*Describe the data collection procedure, including who recorded the data and how.*
Timing and spatial scale	*Indicate the start and stop dates of data collection, noting the frequency and periodicity of sampling and providing a rationale for these choices. If there is a gap between collection periods, state the dates for each sample cohort. Specify the spatial scale from which the data are taken*
Data exclusions	*If no data were excluded from the analyses, state so OR if data were excluded, describe the exclusions and the rationale behind them, indicating whether exclusion criteria were pre-established.*
Reproducibility	*Describe the measures taken to verify the reproducibility of experimental findings. For each experiment, note whether any attempts to repeat the experiment failed OR state that all attempts to repeat the experiment were successful.*
Randomization	*Describe how samples/organisms/participants were allocated into groups. If allocation was not random, describe how covariates were controlled. If this is not relevant to your study, explain why.*

Blinding | *Describe the extent of blinding used during data acquisition and analysis. If blinding was not possible, describe why OR explain why blinding was not relevant to your study.*

Did the study involve field work? ☐ Yes ☐ No

Field work, collection and transport

Field conditions | *Describe the study conditions for field work, providing relevant parameters (e.g. temperature, rainfall).*

Location | *State the location of the sampling or experiment, providing relevant parameters (e.g. latitude and longitude, elevation, water depth).*

Access & import/export | *Describe the efforts you have made to access habitats and to collect and import/export your samples in a responsible manner and in compliance with local, national and international laws, noting any permits that were obtained (give the name of the issuing authority, the date of issue, and any identifying information).*

Disturbance | *Describe any disturbance caused by the study and how it was minimized.*

Reporting for specific materials, systems and methods

We require information from authors about some types of materials, experimental systems and methods used in many studies. Here, indicate whether each material, system or method listed is relevant to your study. If you are not sure if a list item applies to your research, read the appropriate section before selecting a response.

Materials & experimental systems

n/a	Involved in the study	
☐	☒	Antibodies
☒	☐	Eukaryotic cell lines
☒	☐	Palaeontology and archaeology
☐	☒	Animals and other organisms
☒	☐	Clinical data
☒	☐	Dual use research of concern
☒	☐	Plants

Methods

n/a	Involved in the study	
☒	☐	ChIP-seq
☐	☒	Flow cytometry
☒	☐	MRI-based neuroimaging

Antibodies

Antibodies used

For flow cytometry analyses, fluorochrome-conjugated antibodies (Abs) against cell surface antigens were obtained from BD Biosciences (BD), BioLegend (BL), R&D Systems (RD), Miltenyi Biotec (MB), Invitrogen/ThermoFisher (Inv), or eBioscience/ThermoFisher Scientific (eBio). The Abs used are listed below with detailed information in parentheses (clone, Company catalog #, concentration, and lot number).
AF488-anti-mouse CD11c (N418, eBioscience 53-0114-82, 1 µg/mL, B271489)
AF647-anti-mouse CD200 (OX-90, BioLegend 123816, 1 µg/mL, B302597)
AF700-anti-mouse CD116/CSF2RA (698423, R&D Systems FAB6130N, 1 µg/mL, 1636819)
APC eF780-anti-mouse CD14 (Sa14-2, BioLegend 123331, 1 µg/mL, B362596)
APC eFluor 780-anti-mouse Ly-6C (HK1.4, eBioscience 47-5932-82, 1 µg/mL, 2209835)
APC-anti-mouse CD25 (3C7, BioLegend 101910, 1 µg/mL, B276010)
APC-anti-mouse CD301b (URA-1, BioLegend 146813, 1 µg/mL, B376298)
APC-anti-mouse CD88 (20/70, BioLegend 135808, 1 µg/mL, B274277)
AF647-anti-mouse Siglec-F (E50-2440, BD 562680, 1 µg/mL, 8198834)
APC-Cy7-anti-mouse CD45.1 (A20, BioLegend 110716, 1 µg/mL, B228616)
APC-rat IgG2aκ (eBR2a, eBioscience 17-4321-81, 1 µg/mL, 2330525)
BUV395-anti-mouse CD11b (M1/70, BD 565553, 1 µg/mL, 4071716)
BUV395-anti-mouse CD24 (M1/69, BD 744471, 1 µg/mL, 2089786)
BUV395-anti-mouse CD4 (RM4-4, BD 740209, 1 µg/mL, 4207538)
BUV395-anti-mouse CD86 (P03, BD 745716, 1 µg/mL, 1221920)
BUV737-anti-mouse F4/80 (T45-2342, BD 749283, 1 µg/mL, 0062145)
BV510-anti-mouse CD103 (M290, BD 563087, 1 µg/mL, 3268368)
BV510-anti-mouse CD11b (M1/70, BioLegend 101263, 1 µg/mL, B376004)
BV510-anti-mouse CD14 (Sa14-2, BioLegend 123323, 1 µg/mL, B262914)
BV510-anti-mouse CD44 (IM7, BioLegend 103044, 1 µg/mL, B422394)
BV510-anti-mouse CD45.2 (104, BioLegend 109837, 1 µg/mL, B292995)
BV510-anti-mouse Ly-6C (HK1.4, BioLegend 128033, 1 µg/mL, B417848)
BV711-anti-mouse CD200 (OX-90, BD 745548, 1 µg/mL, 1305273)

BV711-anti-mouse CD88 (20/70, BD 743773, 1 μg/mL, 3151022)
BV711-anti-mouse Ly-6A/E (D7, BioLegend 108131, 0.5 μg/mL, B298778)
BV711-anti-mouse Siglec-F (E50-2440, BD 740784, 0.25 μg/mL, 3207519)
BV711-rat IgG2a (RTK2758, BioLegend 400551, 1 μg/mL, B276999)
eFluor450-anti-mouse MHC-II I-Ab (AF6-120.1, eBioscience 48-5320-82, 1 μg/mL, 2731822)
FITC-anti-mouse CD172a (P84, BD 560316, 2.5 μg/mL, 1097523)
FITC-anti-mouse CD40 (3/23, BD 561845, 1 μg/mL, 3237842)
FITC-anti-mouse CD45 (30-F11, BioLegend 103108, 1 μg/mL, B275661)
FITC-anti-mouse CD80 (16-10A1, eBioscience 11-0801-82, 1 μg/mL, E00405-1632)
FITC-anti-mouse CD86 (GL1, BD 561962, 1 μg/mL, 5119857)
FITC-anti-mouse Ly-6C (AL-21, BD 553104, 2.5 μg/mL, 1172671)
FITC-rat IgG2a (R35-95, BD 554688, 1 μg/mL, 67730)
FITC-rat IgMκ (RTK2118, BioLegend 400805, 2.5 μg/mL, B349252)
PE-anti-mouse CD131/CSF2RB (REA193, BD 559920, 1 μg/mL, 3277898)
PE-anti-mouse CD200 (OX-90, BioLegend 123807, 0.5 μg/mL, B280707)
PE-anti-mouse CD301b (URA-1, BioLegend 146804, 1 μg/mL, B274049)
PE-anti-mouse CD3e (145-2C11, BioLegend 100307, 1 μg/mL, B413183)
PE-anti-mouse CD88 (20/70, BioLegend 135806, 1 μg/mL, B409395)
PE-anti-mouse Siglec-F (S17007L, BD 552126, 0.5 μg/mL, B301117)
PE-Dazzle-anti-mouse CD24 (M1/69, BioLegend 101837, 1 μg/mL, B298610)
PE-Dazzle594-anti-mouse F4/80 (BM8, BioLegend 123146, 1 μg/mL, B401902)
PE-rat IgG2a (RTK2758, BioLegend 400508, 0.5 μg/mL, B358742)
PE-rat IgG2b (eB149/10H5, eBioscience 12-4031-82, 0.5 μg/mL, E022489)
PerCP-Cy5.5-anti-mouse CD11c (N418, BioLegend 117328, 1 μg/mL, B396474)
PerCP-Cy5.5-anti-mouse CD45RB (C363-16A, BioLegend 103313, 1 μg/mL, B400338)
PerCP-Cy5.5-anti-mouse CD88 (20/70, BioLegend 135813, 1 μg/mL, B306532)

For transcription factor analysis, cells were permeabilized cells were stained with the following Abs.
eFluo450-anti-mouse FoxP3 (FJK-16s, eBioscience 48-5773-82, 2 μg/mL, 2892548)
APC-anti-mouse GATA3 (W19195B, BioLegend 386908, 0.5 μg/mL, B413492)
PE-anti-mouse HELIOS (22F6, BioLegend 137206, 1 μg/mL, B424503)
BV650-anti-mouse RORgt (Q31-378, BD 564722, 0.5 μg/mL, 4207340)
eFluor450-rat IgG2aκ (eBR2a, eBioscience 48-4321-82, 2 μg/mL, 2258807)
APC-rat IgG2aκ (RTK2758, BioLegend 400511, 0.5 μg/mL, B350155)
PE-anti-hamster IgG (HTK888, BioLegend 400907, 1 μg/mL, B402445)
BV650-anti-mouse IgG1κ (R19-15, BD 744532, 0.5 μg/mL, 9325258)

For naive CD4+ T cells purification, biotinylated antibody cocktail containing the following anti-mouse Abs was used
Biotin-anti-mouse CD11b (M1/70, BD 553309, 0.5 μg/mL, B411021)
Biotin-anti-mouse CD11c (HL3, BioLegend 117304, 0.5 μg/mL, B357263)
Biotin-anti-mouse CD16/32 (2.4G2, BD 553143, 0.5 μg/mL, 8165852)
Biotin-anti-mouse CD19 (6D5, BioLegend 115504, 0.5 μg/mL, B288656)
Biotin-anti-mouse CD25 (PC61, BioLegend 102004, 0.5 μg/mL, B388478)
Biotin-anti-mouse CD44 (IM7, BioLegend 103004, 0.5 μg/mL, B187393)
Biotin-anti-mouse CD49b (DX5, BD 553856, 0.5 μg/mL, 8169517)
Biotin-anti-mouse CD8a (53-6.7, BD 553029, 0.5 μg/mL, 1222937)
Biotin-anti-mouse CD8β (53-5.8, BD 553039, 0.5 μg/mL, 8037991)
Biotin-anti-mouse I-Ab (AF6.120.1, BioLegend 116404, 0.5 μg/mL, B304400)
Biotin-anti-mouse Ly-6C/G (RB6-8C5, BD 553125, 0.5 μg/mL, 9238330)
Purified-anti-mouse CD28 (37.51, BioLegend 102116, 1 μg/mL, B331922)
Purified-anti-mouse CD3e (145-2C11, BioLegend 100331, 1 μg/mL, B398963)

For T cell activation in vitro, following antibodies obtained from BioLegend (BL) were used.
purified anti-mouse CD3e (145-2C11, BL 100331, lot B202622; 1 μg/mL)
purified anti-mouse CD28 (37.51, BL 102116, lot B290928; 1 μg/mL)

For neutralizing EBI3 in DC-T cell coculture experiments we used the antibody obtained from Millipore Sigma anti-EBI3 (V1.4C4.22, SA MABF848).

For Imaging of precision cut lung slices (PCLS), following antibodies were purchased from BD Biosciences (BD) and BioLegend (BL).
AF488-anti-CD324 (DECMA-1, BD 560061, 1 μg/mL, 1924110)
BV605-anti-CD11c (N418, BL 117334; 1 μg/mL, B257897)
PE-anti-F4/80 (BM8, BL 123110; 1 μg/mL, E01705-1637)
APC-anti-CD103 (2E7, BL 121414; 1 μg/mL, B269628)
APC-anti-CD301b (URA-1, BL 146814; 1 μg/mL, B376298)

For cellular indexing of transcriptomes and epitopes sequencing (CITE-Seq), following antibodies were purchased from BioLegend (BL).
CD200 (OX-90, BL 123811; 10 μg/mL, B260039)

	CD301b (URA-1, BL 146817; 10 μg/mL, B275630)<br>Ly6C (HK1.4, BL 128047; 10 μg/mL, B289945)
Validation	All antibodies used in this study are commercially available.<br><br>Antibodies used for flow cytometry are validated by suppliers for the reactivity (mouse) and application (flow cytometry).<br><br>Antibodies used for isolation of naive CD4 T cells were validated by suppliers for reactivity (mouse) using magnet activated cell sorter (MACS). Isolation of naive CD4 T cells using the above listed antibodies was reported in following papers.<br>Naive CD4 T cells (https://doi.org/10.1038/mi.2011.4)<br><br>Information regarding reactivity and application (T cell activation) of anti-mouse CD3e (145-2C11) and CD28 (37.51) antibodies are available in supplier's websites;<br>https://www.biolegend.com/en-us/products/purified-anti-mouse-cd3epsilon-maxpar-ready-antibody-10082<br>https://www.biolegend.com/en-us/search-results/purified-anti-mouse-cd28-maxpar-ready-antibody-10086<br><br>Information regarding reactivity and application (neutralization) of EBI3, anti-EBI3 (V1.4C4.22, SA MABF848), is available on the supplier's website;<br>https://www.sigmaaldrich.com/US/en/product/mm/abt338?<br>srsltid=AfmBOoqzVGG7_yNI382eKbcDYHLeqWtD8qRwiBMnv4tog6R8Z_LDkNFe

Animals and other research organisms

Policy information about studies involving animals; ARRIVE guidelines recommended for reporting animal research, and Sex and Gender in Research

Laboratory animals	C57BL/6J (stock 000664), Cd11cCre (B6.Cg-Tg(Itgax-cre)1-1Reiz/J); stock 008068), CD45.1 (B6.SJL-Ptprca Pepcb/BoyJ; stock 002014), C57BL/6-OT-II TCR transgenic (B6.Cg(TcraTcrb)425Cbn/J; stock 004194), Foxp3eGFP (B6.Cg-Foxp3tm2Tch/J; stock 006772), Ccr7–/– (C57BL/6-Ccr7tm1.1Dnc/J; stock 027913), and Meox2Cre (B6.129S4-Meox2tm1(Cre)Sor/J; stock 003755) mice were purchased from Jackson Laboratories. CD45.1 OT-II mice were bred by crossing CD45.1 and OT-II mice. Foxp3eGFP OT-II mice were bred by crossing Foxp3eGFP and OT-II mice. Csf2fx-ARE mice were generated by Perry Blackshear (NIEHS). The mice lacking 75 bp AU-rich element (ARE) in Csf2 mRNA (Csf2ΔARE) were generated by crossing Meox2Cre and Csf2fx-ARE mice. Csf2rbfx (C57BL/6-Csf2rbtm1c (EUCOMM)Hmgu/Orl) mice originally generated by Burkhard Becher (University of Zürich, Switzerland) were obtained from the European Mouse Mutant Archive. Cd11cCre/wt Csf2rbfx/fx (Csf2rbΔDC) mice were bred by crossing Cd11cCre and Csf2rbfx mice. Mice were bred and housed in specific pathogen-free conditions at the NIEHS with following housing condition; light cycle: 7AM to 7PM, temperature: 72 ± 2 oF, humidity: 40 to 60 %. Mice were used between 6 and 12 weeks of age. All animal procedures complied with institutional guidelines and were approved by the NIEHS Animal Care and Use Committee (Animal Study Protocols 05-22 and 05-23).
Wild animals	Wild animals were not used in this study.
Reporting on sex	Males and females were used in this study as indicated below.<br>Figure 1b: males<br>Figure 1c, d: males<br>Figure 1f: males<br>Figure 2a-e: males<br>Figure 2f, g: females<br>Figure 2h, i: females<br>Figure 3a, b: males and females<br>Figure 3c: males<br>Figure 3d, f: males and females<br>Figure 4a: males<br>Figure 4b-e: males and females<br>Figure 4f: males and females<br>Figure 4g: male<br>Figure 4h: female<br>Figure 5a-c: females<br>Figure 5d: males and females<br>Figure 5e: males<br>Figure 5f: males<br>Figure 6a: males<br>Figure 6c: males<br>Figure 6d: males<br>Figure 7a: females<br>Figure 7b: males<br>Figure 7c: males<br>Figure 7e: males<br>Supplementary Figure 2b: males<br>Supplementary Figure 2c: males<br>Supplementary Figure 2d: males

Supplementary Figure 4c, d: females
Supplementary Figure 5b: males
Supplementary Figure 7b, c: females
Supplementary Figure 8a: males
Supplementary Figure 8b, c: females
Supplementary Figure 8d: males
Supplementary Figure 10b, c, d: males
Supplementary Figure 11b: males

Field-collected samples

No field collected samples were used in this study.

Ethics oversight

All animal procedures complied with institutional guidelines and were approved by the NIEHS Animal Care and Use Committee (Animal Study Protocols 05-22 and 05-23).

Note that full information on the approval of the study protocol must also be provided in the manuscript.

Plants

Seed stocks

Report on the source of all seed stocks or other plant material used. If applicable, state the seed stock centre and catalogue number. If plant specimens were collected from the field, describe the collection location, date and sampling procedures.

Novel plant genotypes

Describe the methods by which all novel plant genotypes were produced. This includes those generated by transgenic approaches, gene editing, chemical/radiation-based mutagenesis and hybridization. For transgenic lines, describe the transformation method, the number of independent lines analyzed and the generation upon which experiments were performed. For gene-edited lines, describe the editor used, the endogenous sequence targeted for editing, the targeting guide RNA sequence (if applicable) and how the editor was applied.

Authentication

Describe any authentication procedures for each seed stock used or novel genotype generated. Describe any experiments used to assess the effect of a mutation and, where applicable, how potential secondary effects (e.g. second site T-DNA insertions, mosiacism, off-target gene editing) were examined.

Flow Cytometry

Plots

Confirm that:

- [x] The axis labels state the marker and fluorochrome used (e.g. CD4-FITC).
- [x] The axis scales are clearly visible. Include numbers along axes only for bottom left plot of group (a 'group' is an analysis of identical markers).
- [x] All plots are contour plots with outliers or pseudocolor plots.
- [x] A numerical value for number of cells or percentage (with statistics) is provided.

Methodology

Sample preparation

For mouse lung dendritic cells: Lungs were perfused and harvested at indicated time points post-allergic sensitization or at steady state conditions. Lungs were minced and digested using enzymes Liberase TM (100 ug/mL), Collagenase XI (250 ug/mL), Hyaluronidase (1 mg/mL), and DNase I (200 ug/mL) for 1 hr. After digestion, a single cell suspension was created by passing the tissue through a 70 um strainer and low density cells for DC enrichment were collected on a 16% Nycodenz gradient.

For mouse lung T cells: Lungs were perfused and harvested at the indicated time points post-sensitization or at steady state conditions. Lungs were minced and digested with the same enzymes for 30 minutes. After digestion, low density cells were collected on a histopaque 1083 gradient.

For bone marrow cells: tibias, femurs, humeri, and sternum bones were collected from mice. The bones were crushed and red blood cells lysed with ACK buffer containing 0.15 M ammonium chloride and 1 mM potassium bicarbonate. A single cell suspension was created using a 70 um strainer. Total bone marrow cells were then cultured in complete RPMI-10 media containing either FLT3L (100 ng/mL) or FLT3L (100 ng/mL) and GM-CSF (10 ng/mL).

For mediastinal lymph nodes: lymph nodes were minced and digested using enzymes Liberase TM (100 ug/mL), Collagenase XI (250 ug/mL), Hyaluronidase (1 mg/mL), and DNase I (200 ug/mL) for 30 minutes. After digestion, a single cell suspension was created by passing the tissue through a 70 um strainer, and red blood cells lysed using ACK buffer containing 0.15 M ammonium chloride and 1 mM potassium bicarbonate.

Cells were diluted to one million cells/100 µL and incubated with a non-specific binding blocking reagent cocktail of anti-mouse CD16/CD32 (2.4G2), normal mouse and rat serum (Jackson ImmunoResearch). Cell surface antigens were stained with fluorochrome antibodies.

Instrument

For cell analysis, LSR-Fortessa (BD Biosciences) with 5 lasers. For cell purification, FACS Aria-II (BD Biosciences) with 5 lasers.

Software	Data collection: FACS Diva version 8.0.1 and 8.0.2 (BD)<br>Data analysis: Flow Jo versions 10.8.1 and 10.8.2 (BD)
Cell population abundance	Stopping gate setting was 10,000 cells in cDC2 gate in Diva software for analyses.<br>The purify of post-sort fractions (>90%) was verified by re-analysis in flow cytometry.
Gating strategy	Gating strategy for purifying lung cDC1 and cDC2 subsets for Fig. 1c and f are depicted in Extended Data Fig. 1a. cDC1s: CD11c+I-A+CD11b–CD103+CD14–CD88–SiglecF–F4/80– singlets. cDC2s: CD11c+I-A+CD11b+CD103–CD88–SiglecF–F4/80– singlets.<br><br>Gating strategy for measuring Treg differentiation in Fig. 1c and d, 4g, 5e, and Extended Data Fig. 8d is depicted in Extended Data Fig. 1b. Tregs were CD4+CD3e+CD44+I-A–Live/Dead– singlets.<br><br>Gating strategy for cDC2 subset purification used for Fig. 1d and Extended Data Fig. 2c and d is depicted in Extended Data Fig. 1c. CD11c+I-A+CD11b+CD14–CD88–SiglecF–F4/80– singlets.<br><br>Gating strategy for measuring Treg transcription factors in Extended Data Fig. 2b is depicted in Extended Data Fig. 2a. Tregs were FoxP3+CD4+CD3e+CD44+I-A–Live/Dead– singlets.<br><br>Gating strategy for scRNA-sequencing for Fig. 2b-d, f are depicted in Extended Data Fig. 3a. Total cDC2s were CD45+CD11c+I-A+CD11b+CD103–CD88–SiglecF–F4/80–Live/Dead– singlets.<br><br>Gating strategy for flow cytometric analysis of cDC1 and cDC2 subsets use for Fig. 2f-g depicted in Extended Data Fig. 4a. cDC1s: CD11c+I-A+CD103+CD11b–CD14–CD88–SiglecF–F4/80– singlets. cDC2s: CD11c+I-A+CD11b+CD103–CD88–SiglecF–F4/80– singlets.<br><br>Gating strategy for cDC2 subset sorting for adoptive transfer in Fig. 2h and Extended Data Fig. 4c depicted in Extended Data Fig 4b. CD11c+I-A+CD11b+CD103–CD88–SiglecF–F4/80– singlets.<br><br>Gating strategy for flow cytometric analyses of migratory cDC2s in Fig 3a-b and Extended Data Fig. 5b are depicted in Extended Data Figure 5a. CD11c+I-A+CD11b+PKH+CD88–CD3e–CD19– singlets.<br><br>Gating strategy for Treg analysis used in Fig. 3c and d is depicted in Extended Data Fig. 4c. CD4+CD3e+CD44+CD45.1+I-A–Live/Dead– singlets.<br><br>Gating strategy for flow cytometry analysis of lung cDC2s from Csf2 and Csf2rb mice used for Fig. 4c-f, and Fig. 5a-d depicted in Extended Data Fig. 7a. cDC1s: CD11c+I-A+CD103+CD11b–CD88–SiglecF–F4/80– singlets. cDC2s: CD11c+I-A+CD11b+CD103–CD88–SiglecF–F4/80–Live/Dead– singlets.<br><br>Gating strategy for flow cytometry analysis of lung cDC2 subsets used for Fig. 6a depicted in Extended Data Fig. 9a. cDC2s: CD11c+I-A+CD11b+CD88–SiglecF–F4/80–Live/Dead– singlets.<br><br>Gating strategy for Treg analysis used for Fig. 6c, d and Extended Data Fig. 10b-d depicted in Extended Data Fig. 10a. CD4+CD3e+CD44+I-A–Live/Dead– singlets.<br><br>Gating strategy for BMDC2 analysis used for Fig. 7a and Extended Data Fig. 11b are depicted in Extended Data Fig. 11a. CD11c+I-A+CD172a+CD24–Ly6C–CD88–Live/Dead– singlets.<br><br>Gating strategy for purifying BMDC2 subsets in Fig. 7b, c, and e depicted in Extended Data Fig. 11c. CD11c+I-A+CD172a+CD24–CD88–Live/Dead– singlets.<br><br>Gating strategy for flow cytometric analysis of Tregs following BMDC ex vivo culture used for Fig. 7c depicted in Extended Data Fig. 11d. CD4+CD3e+CD44+I-A–Live/Dead– singlets.

☒ Tick this box to confirm that a figure exemplifying the gating strategy is provided in the Supplementary Information.
